# Supplementary material for: Deep learning-based incoherent holographic camera enabling acquisition of real-world holograms for holographic streaming system
Source: Nat Commun. 2023 Jun 14;14:3534. doi: 10.1038/s41467-023-39329-0 (PMC10267150; doi:10.1038/s41467-023-39329-0)
Supplement: Supplementary file 1 — Supplementary Information [file 41467_2023_39329_MOESM1_ESM.pdf]

(Supplementary Information) Deep learning-based incoherent holographic camera enabling acquisition of real-world holograms for holographic streaming system

1 Overview of the GPSIDH system

In this section, we briefly review the principles of the GPSIDH system. The GPSIDH system induces self-interference in the light waves originating from the point sources via the GP lens and acquires complex holograms based on a four-phase shifting technique using a polarized sensor, which provides intensity maps for the four different polarization states. Fig. S1 presents a schematic diagram of the GPSIDH system.

The GP lens is a polarization-selective wavefront devision device made of photoaligned liquid crystal layers [1]. The topological state of the polarization determined by the molecular anisotropy of the liquid crystal induces phase modulation, unlike the conventional phase modulation achieved using the birefringence of the liquid crystal. The GP lens acts as either a convex or a concave lens based on the circular polarization state of the incident light and induces a phase shift. Additionally, it operates over the entire visible light range; therefore, the GPSIDH system has been demonstrated as a full-color holographic video system [2].

1.1 Hologram formation

The impulse response function is first derived for a single point source, and the formulation is then extended to a 3D object.

1.1.1 Hologram formation from a single point source

The light originating from a point source first passes through the linear polarizer and is then transmitted through the GP lens. The GP lens can be considered a stack of two lenses with focal lengths of f and $-f$; it induces converging and diverging beams. These two waves can be described as waves originating from two point source images at locations, z_p and z_n, based on the

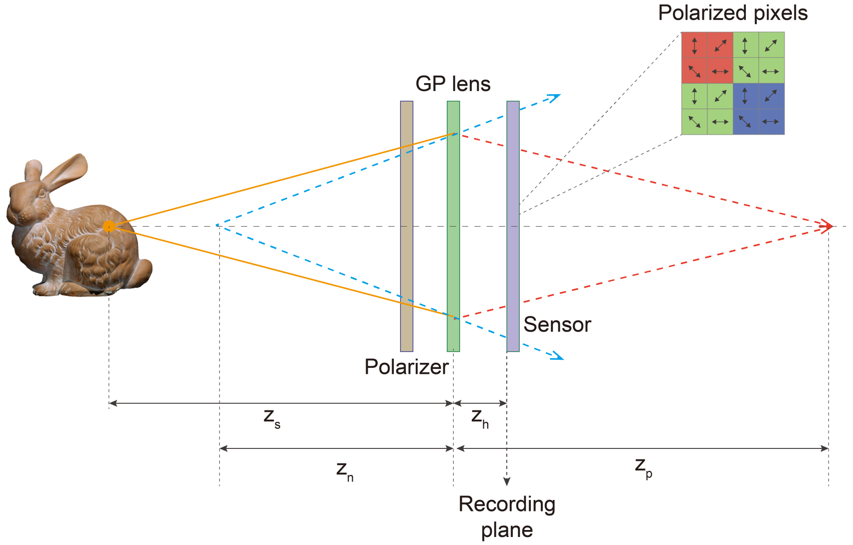

Fig. S1 Configuration of the GPSIDH system.

lens equation:

$$z_p = \frac{z_s f}{z_s - f}, \; z_n = \frac{z_s f}{z_s + f}. \tag{S1}$$

Here z_s denotes the distance between the source object and the GP lens. Despite their mathematical descriptions, those two waves actually originate from the same point source, thus they interfere at the sensor plane within the temporal coherence range, forming a Fresnel zone pattern. The exact form of the interference pattern can be derived as follows. For a point source with complex amplitude A_0 positioned at $r_s = (x_s, y_s)$ in the xy-plane, the optical field obtained after propagating distance z is given as

$$\mathbf{U}(x, y, z;\; x_s, y_s) = \frac{\mathbf{A}_0}{z} e^{ikz} e^{i\pi \frac{x^2+y^2}{z\lambda}} e^{-i\frac{2\pi}{z}(x_s x + y_s y)}. \tag{S2}$$

The quadratic phase term describes the spherical wave, and the linear phase term considers the position of the point source. With respect to the sensor plane that is positioned z_h behind the GP lens, two point-sources are placed at $z_p - z_h$ and $z_n + z_h$ in depth and their spatial positions in the xy-plane are $(M_p x_s, M_p y_s)$ and $(M_n x_s, M_n y_s)$ where

$$M_p = \frac{-f}{z_s - f}, \; M_n = \frac{-f}{z_s + f}.$$

due to the lens magnification. Then, the optical field $\mathbf{E}_c$ at the camera sensor plane can be formulated as

$$\begin{aligned}\mathbf{E_c}(x, y;\; x_s, y_s, z_s) =& \mathbf{U}(x, y, z_p - z_h;\; M_p x_s, M_p y_s) e^{i\delta/2} \\ &+ \mathbf{U}(x, y, z_n + z_h;\; M_n x_s, M_n y_s) e^{-i\delta/2}.\end{aligned} \tag{S3}$$

Here, the additional phase terms $\delta/2$ and $-\delta/2$ are introduced due to the GP lens [2]. Setting $\psi_p = \mathbf{U}(x, y, z_p - z_h;\ M_p x_s, M_p y_s)$ and $\psi_n = \mathbf{U}(x, y, -(z_n + z_h);\ M_n x_s, M_n y_s)$, the intensity map I_c at the camera sensor plane can be formulated as

$$I_c(x, y;\ x_s, y_s, z_s) = |\mathbf{E}_c|^2 = |\psi_p e^{i\delta/2} + \psi_n e^{-i\delta/2}|^2. \tag{S4}$$

Capturing I_c using conventional 2D cameras does not provide sufficient information to reconstruct the optical field $\mathbf{E}_c$. The combinations of the linear polarizer and single-shot polarization-dependent acquisition at the polarized sensor produce four different geometric phases, $\delta = 0, \pi/2, \pi, 3\pi/2$ [2], yielding phase-shifting images as follows:

$$I_{c,0} = \psi_p\psi_p^* + \psi_n\psi_n^* + \psi_p\psi_n^* + \psi_p^*\psi_n \tag{S5}$$
$$I_{c,1} = \psi_p\psi_p^* + \psi_n\psi_n^* + i\psi_p\psi_n^* - i\psi_p^*\psi_n \tag{S6}$$
$$I_{c,2} = \psi_p\psi_p^* + \psi_n\psi_n^* - i\psi_p\psi_n^* - \psi_p^*\psi_n \tag{S7}$$
$$I_{c,3} = \psi_p\psi_p^* + \psi_n\psi_n^* - i\psi_p\psi_n^* + i\psi_p^*\psi_n. \tag{S8}$$

Then the complex hologram $\mathcal{H}_i$ is reconstructed from those four phase-shifting images as follows:

$$\begin{aligned}\mathcal{H}_i &= (I_{c,0} - I_{c,2}) - i(I_{c,1} - I_{c,3}) \propto \psi_p\psi_n^* \\ &= \mathbf{U}(z_p - z_h, M_p r_s)\mathbf{U}^*(-z_n - z_h, M_n r_s) \\ &\propto e^{i\pi\frac{x^2+y^2}{z_r\lambda}} e^{i\frac{2\pi}{z_r}(M' x_s x + M' y_s y)}\end{aligned} \tag{S9}$$

where

$$z_r = \frac{(z_n + z_h)(z_p - z_h)}{(z_n + z_p)},\quad M' = \frac{M_p(z_n + z_h) - M_n(z_p - z_h)}{(z_n + z_p)}. \tag{S10}$$

The hologram $\mathcal{H}_i$ is the impulse response function for the original physical point source placed at (x_s, y_s, z_s), which effectively represents the spherical wave originating from the *virtual* point source positioned at $(M' x_s, M' y_s, z_r)$. This implies that the captured hologram encodes the converted depth of real-world scenes instead of the actual physical depth.

When the target object is placed at the physical depth z_s, the image must be reconstructed at z_r using d-ASM according to Eq. (S10). Only the physical depths are mentioned in the main manuscript to avoid any confusion.

1.1.2 Hologram formation from 3D objects

A 3D object can be described as the collection of point sources, and their contributions are incoherently summed up under incoherent illumination. Therefore,

the intensity map at the sensor for an arbitrary 3D scene can be described as follows:

$$I(x, y) = \iiint I_s(x_s, y_s, z_s) I_c(x, y;\ x_s, y_s, z_s)\ dx_s dy_s dz_s \tag{S11}$$

where $I_s(x_s, y_s, z_s)$ represents the intensity of the point source at (x_s, y_s, z_s). Again, the acquisition of this intensity map using the polarized sensor produces four phase-shifting images as follows:

$$I_k(x, y) = \iiint I_s(x_s, y_s, z_s) I_{c,k}(x, y;\ x_s, y_s, z_s)\ dx_s dy_s dz_s \tag{S12}$$

where $k = 0$, 1, 2, 3. Finally, the hologram $\mathcal{H}$ of the 3D object is reconstructed as follows:

$$\begin{aligned} \mathcal{H} &= (I_0 - I_2) - i(I_1 - I_3) \\ &= \iiint I_s(x_s, y_s, z_s) \mathcal{H}_i(x, y;\ x_s, y_s, z_s)\ dx_s dy_s dz_s. \end{aligned} \tag{S13}$$

1.2 Image quality degradation

Severe image quality degradation, such as color mismatches, contrast reductions, and scattered noise patterns, can be observed in the GPSIDH system. In this section, we discuss the various sources of noise.

The spatial variance of the impulse response functions is the primary cause of image degradation. Figs. S2b and S2e present the real parts of the impulse response functions for LED 1 (Fig. S2a) and LED 2 (Fig. S2d) captured by our GPSIDH setup, respectively. LED 1 is positioned at the center of the field of view (FoV), and the measured impulse response function fills up the entire sensor plane. The captured Fresnel zone pattern is shifted accordingly for the off-axis point source (LED 2), which has the same brightness as that of LED 1. A degraded diffraction efficiency is observed for LED 2, as both holograms are normalized by the same maximum value. If the focal images are reconstructed and normalized by their own maximum intensities, clear focuses can be obtained for both cases (Figs. S2c and f). A problem arises when two LEDs are captured simultaneously (Fig. S2f). The signal in the captured hologram (Fig. S2h) is dominated by the signal from LED 1, as the diffraction efficiency is lower for the off-axis point source. This results in a lower intensity for LED 2 in the reconstructed focal image (Fig. S2i). Based on this observation, image degradation toward the outer edge of the FoV can be expected in general cases. To confirm this, for a grid test pattern (Fig. S2j), the corresponding hologram (Fig. S2k) is captured, and the focal image (Fig. S2l) is reconstructed. It can be easily determined that the signal degrades in the boundaries where the outer rows and columns are almost invisible. Resolving this spatial variance of the impulse response functions is particularly difficult because they cannot be

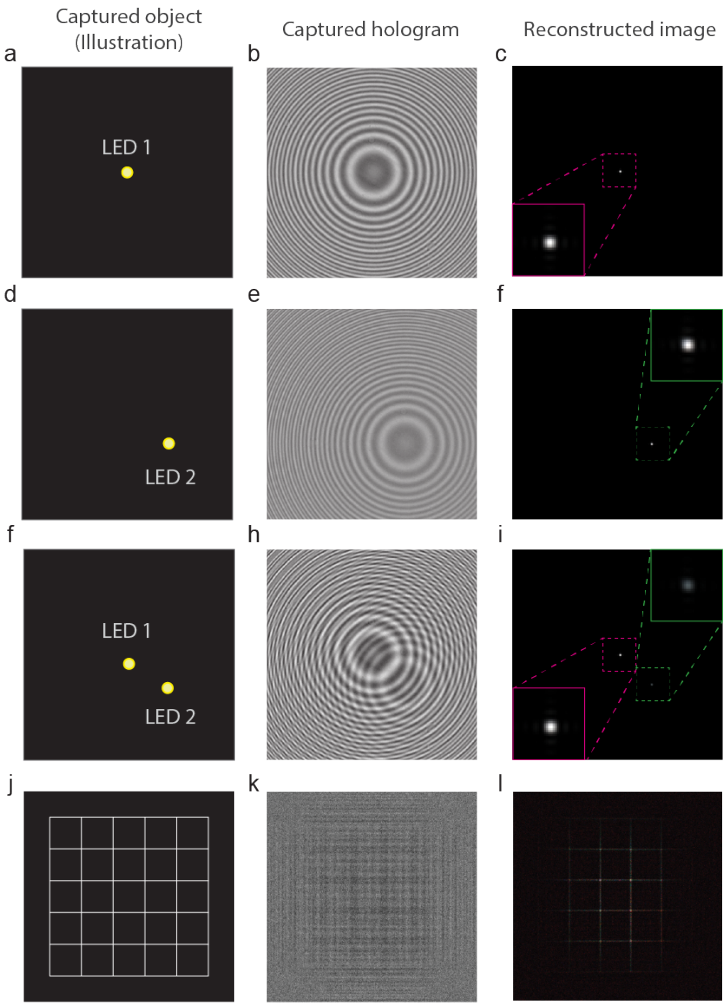

Fig. S2 Image degradation of the GPSIDH system. a, d, f Illustrations of various LED configurations and **j** a grid pattern displayed on a 2D monitor for testing image degradation. **b, e, h, k** Captured holograms and **c, f, i, l** reconstructed focal images. All captured holograms are plotted for the real part of the green channel. The holograms of the LEDs are normalized by the same magnitude in **b, e, h**. The reconstructed images are normalized by the maximum intensity of each image.

compensated by using a simple calibration map. The contributions from multiple point sources are intertwined in a content-dependent manner; therefore, compensating for the spatial variance of the system is not straightforward.

GP lens aberrations form another cause of image quality degradation. The GP lens is fabricated by the photoalignment of liquid crystal layers using the holographic interference technique. In this process, the lens is designed to be a half-wave retarder for achieving maximum efficiency at a particular wavelength, which induces chromatic aberrations [3, 4]. The wavelength dependency

of the GP lens also affects its phase modulation efficiency and focal length. Incomplete phase modulation produces an additional bias noise, and the focal length variance changes the reconstruction distance based on the wavelength.

The shot noise of the polarized sensor contributes to the system noise, and the limited light efficiency induced by the polarization filters also results in a low SNR. Moreover, the presence of high-reflectance materials deteriorates the neighboring signals and effectively reduces the SNR as the impulse response functions from multiple point sources are overlapped and incoherently summed up.

One possible approach for solving the aforementioned problems involves modeling the imperfections of the impulse response functions, the aberrations of the GP lens, camera noise, and camera-specific calibration parameters based on recently developed camera-in-the-loop approaches [5–8], which have successfully demonstrated exceptional quality improvements on displayed holograms. Instead, we approach tackling the image degradation issue by using a fully convolutional neural network, expecting that the spatially variant features of impulse response functions and hardware-specific calibrations can be learned and handled by the network. Conceptually, we propose that a neural network can be used as a postprocessing filter for hologram data, as many image processing filters are available for 2D images.

1.3 Matching the captured hologram to the target image

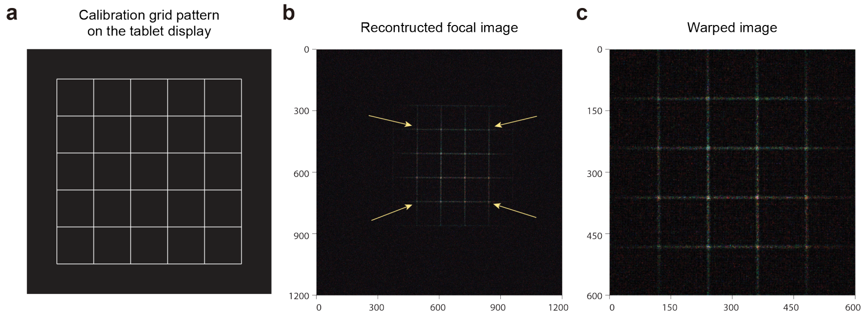

Fig. S3 Calibration process. a Displayed calibration pattern on the tablet display and **b** reconstructed focal image at the depth of the display. **c** Focal image warped to the rectangular area with a resolution of 600 × 600.

In this section, we describe a procedure for matching the captured hologram and the target image displayed on the tablet display. Our initial experiments indicate that the effective ROI of the system is smaller than 600 × 600. The image quality already significantly drops outside the 400 × 400 region in the center, but we aim to enhance the image quality up to 600 × 600. At each depth, we display a 5 × 5 grid pattern as the calibration pattern on the tablet display, as shown in Fig. S3a. We adjust the size of the grid pattern so that the

size of the whole pattern in the reconstructed focal image (Fig. S3b) is approximately 600 × 600 pixels. Therefore, we observe a larger calibration pattern when the display is placed farther from the camera. Due to image degradation, it is difficult to locate the four outermost corners of the calibration image in the focal image. Thus, we locate the positions of the four inner corners (indicated by yellow arrows) and extrapolate the positions of the four outermost corners. Using this information, we warp the calibration pattern image to a 600 × 600 rectangular area, as shown in Fig. S3c. We perform homography warping using Kornia, which is a differential library for PyTorch. When we capture holograms for the training dataset, high-resolution target images are resized to the same size as the calibration pattern and displayed inside the area corresponding to the calibration pattern. Then, the final warped images derived from the captured holograms are compared to the resized target images with resolutions of 600 × 600 to compute the image loss.

2 System parameters of the GPSIDH system

In this section, we theoretically derive the lateral and axial resolutions per the GP overlap conditions. Then, we also experimentally confirm them based on our system parameters. Finally, we derive the field of view (FoV) of the GP-SIDH system. Before we discuss the lateral and axial resolutions, we briefly show that the GP-SIDH system cannot be configured to have the perfect beam overlap condition. It should not impact our system design because we intentionally use the partial beam overlap condition to increase the FoV.

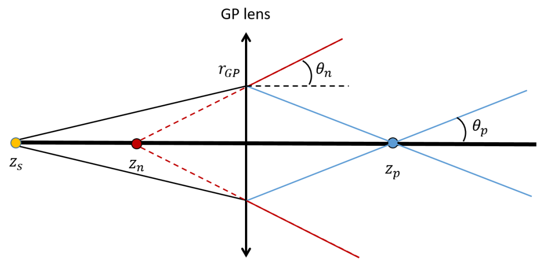

Fig. S4 Image formation in the GP-SIDH system.

Fig. S4 shows the image formation diagram of the GP-SIDH system. Here, z_s denotes the distance between the source object and the GP lens; r_p represents the radius of the holograms; and z_p and z_n denote the locations of the virtual images formed by the positive and negative focal lengths of the GP lens, respectively. In order to satisfy the perfect overlap condition as in FINCH, the propagation angle θ_p induced by the positive focal length should be greater than the propagation angle θ_n generated by the negative focal length.

$$\theta_p - \theta_n > 0 \tag{S14}$$

where

$$\theta_p = \arctan(r_{GP}/|z_p|), \theta_n = \arctan(r_{GP}/|z_n|), \tag{S15}$$

Therefore, we can check the following relationship instead:

$$\frac{r_{GP}}{|z_p|} - \frac{r_{GP}}{|z_n|} > 0. \tag{S16}$$

According to the lens formula, we have

$$z_p = \frac{f_{GP}z_s}{z_s - f_{GP}}, z_n = \frac{-f_{GP}z_s}{z_s + f_{GP}}. \tag{S17}$$

Then, we obtain the following relationship:

$$\frac{r_{GP}}{|z_p|} - \frac{r_{GP}}{|z_n|} = \frac{r_{GP}}{f_{GP}z_s}(|z_s - f_{GP}| - |z_s + f_{GP}|). \tag{S18}$$

Since both z_s and f_{GP} are positive values, $r_{GP}/|z_p| - r_{GP}/|z_n| < 0$ and thus $\theta_p - \theta_n < 0$. Therefore, the perfect overlap condition cannot be met with the GP-SIDH systems. In the following sections, we consider only the partial overlap condition in our GP-SIDH system.

2.1 Theoretical derivation of lateral and axial resolutions of the GP-SIDH system

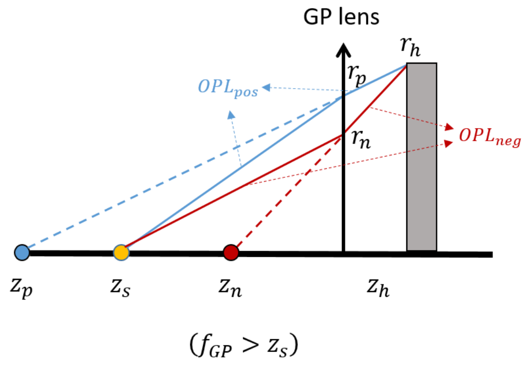

Fig. S5 System configuration of the GP-SIDH system.

Here, we theoretically derive the lateral and axial resolution of the GP-SIDH system. The representative system configuration is shown in Fig. S5. In our GP-SIDH system, $f_{GP} = 1000$ mm and the target depth range is $z_s \in$[300 mm, 480 mm]; therefore, we consider the case when $f_{GP} > z_s$. In conventional

digital holography, the effective lateral and axial resolutions are given as follows [9]

$$R_{lateral} = 0.61\frac{\lambda}{NA \cdot M_T}, \tag{S19}$$

$$R_{axial} = 2\frac{\lambda}{NA^2 \cdot M_A}. \tag{S20}$$

where M_T is the transverse magnification; M_A is the axial magnification, and NA is the numerical aperture (NA) of the system. The NA of the captured incoherent hologram is

$$NA = \frac{r_h}{z_r} \tag{S21}$$

where r_h is the hologram radius and z_r is the reconstruction distance (not shown in Fig. S5) which is given as

$$z_r = \frac{(f_{GP}(z_s + z_h))^2 - (z_s z_h)^2}{2 f_{GP} z_s^2}. \tag{S22}$$

Here, z_h denotes the distance between the GP lens and the image sensor. Therefore, the detailed form of the resolutions can be expressed as

$$R_{lateral} = 0.61\frac{\lambda}{NA \cdot M_T} = 0.61\frac{\lambda z_r}{r_h}\frac{z_s}{z_h}, \tag{S23}$$

$$R_{axial} = 2\frac{\lambda}{NA^2 \cdot M_A} = \frac{\lambda z_r^2}{r_h^2}\frac{z_s^3}{z_h f_{GP}(z_s + z_h)}. \tag{S24}$$

As the beam overlap is proportional to z_h, we study the dependence of the lateral and axial resolutions on z_h. We set z_s to 390 mm, which is the center distance of the depth range of DeepIHC. Then, the corresponding reconstruction depth z_r becomes 530 mm. In order to fully determine the axial and lateral resolutions, we need to compute the hologram radius r_h, which can be determined using the following criterion:

$$r_h = \min(r_{OPL}, r_{Nyquist}, r_{CMOS}). \tag{S25}$$

Here, $r_{OPL}, r_{Nyquist}, r_{CMOS}$ represent the maximum hologram radius limited by optical path difference, sensor sampling frequency, and size of CMOS sensor, respectively.

Firstly, r_{OPL} is determined by the interference formation condition as shown in Fig. S5.

$$OPD = \Delta OPL = |OPL_{pos} - OPL_{neg}| < \text{(coherence length)} = \frac{\lambda^2}{\Delta\lambda}. \tag{S26}$$

For a given r_{OPL}, the corresponding hologram radius r_p and r_n at the GP lens are given as

$$r_p = \frac{z_p}{z_p + z_h} r_{OPL}, r_n = \frac{z_n}{z_n + z_h} r_{OPL}, \tag{S27}$$

where z_p and z_n denote the locations of the virtual images formed by the positive and negative focal lengths of the GP lens, respectively.

$$z_p = \frac{f_{GP} z_s}{z_s - f_{GP}}, z_n = \frac{-f_{GP} z_s}{z_s + f_{GP}}. \tag{S28}$$

Therefore, the optical path difference can be derived using a simple geometric consideration:

$$\begin{aligned} OPD =& [(z_s^2 + r_p^2)^{\frac{1}{2}} + (z_h^2 + (r_{OPL} - r_p)^2)^{\frac{1}{2}}] - \\ & [(z_s^2 + r_n^2)^{\frac{1}{2}} + (z_h^2 + (r_{OPL} - r_n)^2)^{\frac{1}{2}}] \leq \frac{\lambda^2}{\Delta\lambda}. \end{aligned} \tag{S29}$$

r_{OPL} is the maximum value that satisfies the condition in Eq. (S29). Assuming the illumination wavelength of 550 nm, the spectral width of 100 nm of our system provides r_{OPL} of 65 mm. However, this derivation assumes that r_p and r_n are not limited by the aperture size of the GP lens. In case the GP lens is a limiting factor, r_{OPL} is computed using the following formula:

$$r_{OPL} = r_{GP} \frac{f_{GP} z_s + f_{GP} z_h - z_h z_s}{f_{GP} z_s}. \tag{S30}$$

The GP lens used in our system has a 2-inch diameter; therefore, the aperture size of the GP lens is the limiting factor of r_{OPL} and the final value is 26 mm.

Secondly, $r_{Nyquist}$ describes the limitation posed by the sampling rate of the image sensor, which can be derived from the following relationship between the pixel pitch Δx of the image sensor and central wavelength λ:

$$r_{Nyquist} = z_r \frac{\lambda}{\Delta x}. \tag{S31}$$

The center wavelength of 550 nm and the pixel pitch 3.45 μm of our system provides $r_{Nyquist}$ of 40 mm.

Lastly, r_{CMOS} = 7 mm in our GP-SIDH system, and the final hologram radius is determined as

$$r_h = \min(r_{OPL}, r_{Nyquist}, r_{CMOS}) = \min(26mm, 40mm, 7mm) = 7mm. \tag{S32}$$

Therefore, the hologram radius is currently limited by the size of the image sensor. This indicates that the GP-SIDH system has room for enhancing lateral and axial resolutions by employing the synthetic aperture strategy.

Using the computed hologram radius, we obtain the effective lateral and axial resolutions as a function of z_h in Fig. S6. As the distance z_h between the GP lens and image sensor increases, the beam overlap also increases. The result shows that as the beam overlap increases, the lateral and axial resolutions increase as well. However, the increased z_h leads to the reduced FoV (see Sec.

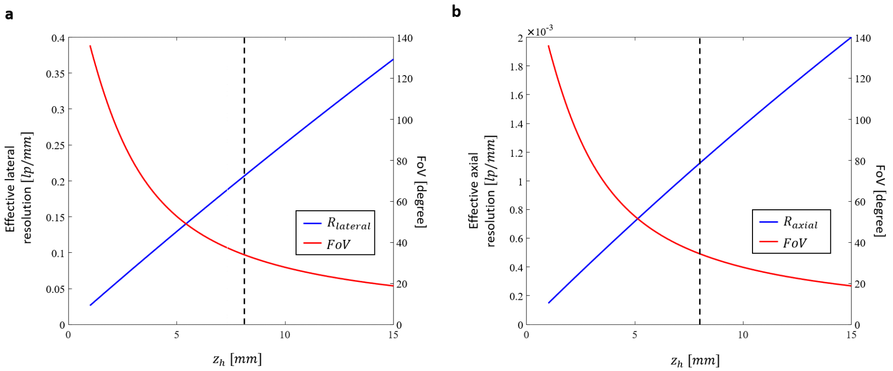

Fig. S6 Dependence of the lateral and axial resolutions on z_h. a Effective lateral resolution and FoV as a function of z_h. **b** Effective axial resolution and FoV as a function of z_h.

2.3). We use lp/mm unit to show the opposite trends of the resolutions and FoV. Therefore, we use z_h of 8 mm (indicated with the black dashed line) in our system as a compromise between the FoV and resolutions. For this setting, the lateral and axial resolutions are calculated as 2.5 mm and 444 mm in our system. In the following section, we also experimentally confirm the computed resolution values. We found that the axial resolution seems to be large, however, our analysis shows that the large axial resolution does not imply that objects that are separated by a distance smaller than the axial resolution cannot be differentiated. We can still observe a clear defocus effect within the depth range that is smaller than the axial resolution; therefore, we discuss the implication of the axial resolution in the context of 3D imaging.

2.2 Experimental confirmation of lateral and axial resolutions of the GP-SIDH system

2.2.1 Lateral resolution

In order to validate the theoretical values of the lateral resolution, we conducted two experiments using two point sources and resolution targets. The theoretical derivation on the lateral resolution is based on the point spread functions of the point light sources. To experimentally reproduce this setting, we generate two point sources and set the lateral position difference using a beam splitter. We set three different lateral separation values of 0 mm, 2 mm, and 3 mm and reconstruct the images and corresponding intensity profiles for each case, as shown in Fig. S7. We found that the lateral separation of 2 mm exhibits the intensity profile (Fig. S7e) similar to the Rayleigh criterion, and the lateral separation of 3 mm (Fig. S7f) indicates that two point sources are already well resolvable. Therefore, we can conclude that the Rayleigh resolution is approximately 2 mm, which well matches the theoretical value of 2.5 mm. We also confirmed the lateral resolution by imaging the USAF resolution

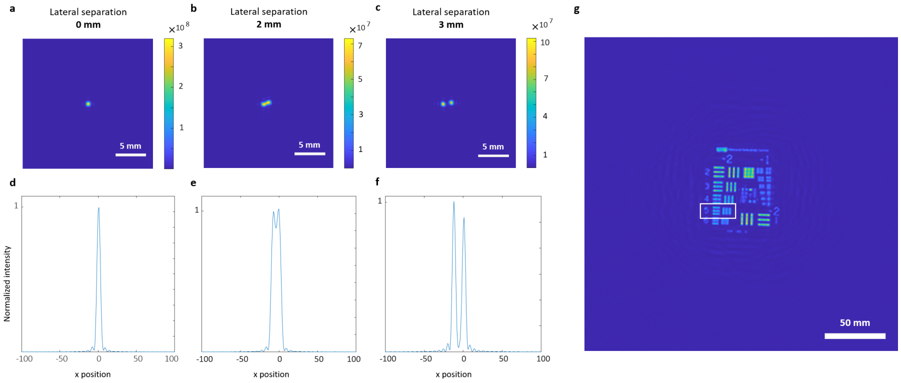

Fig. S7 Lateral intensity profiles of the two point sources for various lateral separations. a, b, c Reconstructed images of the two point sources when the separation was set to 0 mm, 2 mm, and 3 mm, respectively, and **d, e, f** corresponding intensity profiles along the x-axis. g Reconstructed image of the hologram that captures the USAF resolution target. The minimum resolvable pattern is indicated by a white rectangle.

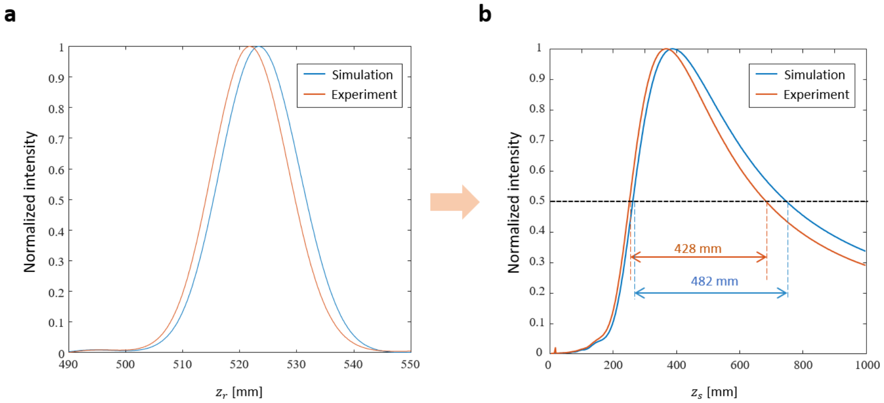

Fig. S8 Axial intensity profiles of a point source. a Axial intensity profiles of the simulated and measured holograms of a point source. The profiles are derived as a function of the reconstruction distance z_r. **b** Axial intensity profiles expressed in terms of the physical distance z_s.

target, as shown in Fig. S7g. The minimum resolvable pattern indicated by the white rectangle was 0.353 lp/mm, which is converted to a lateral resolution of 2 mm.

2.2.2 Axial resolution

We measure the axial resolution based on the full width at half maximum (FWHM) of the axial profiles of point light sources, following the approach in Ref. [10]. We captured the hologram of a point source placed at $z_s = 390$ mm and reconstructed the axial intensity profiles (orange solid line) as a function

of z_r, as shown in Fig. S8a. We also simulated a hologram for the point source placed at the same position and acquired an almost identical axial intensity profile (blue solid line) in Fig. S8a. We express both intensity profiles as a function of z_s in Fig. S8b. The FWHM of the experimental and simulated results are 428 mm and 482 mm, respectively. Therefore, we see that the validation results of the experiment and simulation match reasonably well with the theoretical axial resolution of 444 mm.

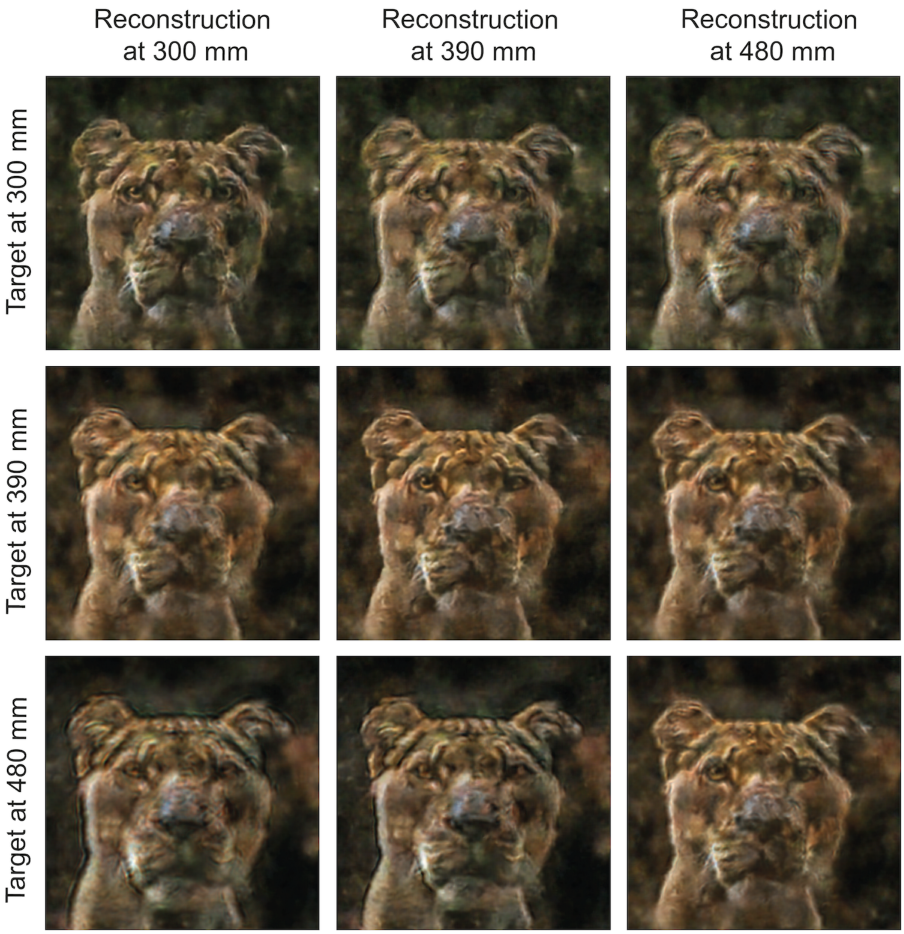

Fig. S9 Reconstructed images from the captured holograms at various depths. DeepIHC holograms are acquired for the target at 300 mm (Top), 390 mm (Middle), and 480 mm (Bottom) and images are reconstructed from the holograms at three different reconstruction depths at 300 mm (Left), 390 mm (Center), and 480 mm (Right).

2.2.3 Implication of axial resolution in the context of 3D camera

Although we confirmed the good match between the theoretical predictions and experimental results, we found that the axial resolution of 444 mm seems to be large compared to the depth range of the DeepIHC which is 180 mm. We note that the axial resolution of the system does not fully describe the important aspect of the 3D camera system: the amount of the defocus blur. When we use the GP-SIDH system for daily-use cameras and show acquired holograms on holographic displays, the essential role of the captured holograms is to provide the visually noticeable defocus effect rather than to enable the quantification of the exact axial separation between the objects. We believe our system provides such a defocus capability as demonstrated in Fig. S9. We placed the same image at three different depths, namely, 300 mm, 390 mm, and 480 mm, and acquired the corresponding holograms using DeepIHC. The top, middle, and bottom rows present reconstructed images from the DeepIHC holograms of the target at 300 mm, 390 mm, and 480 mm, respectively. Although the depth separation between targets is well below the axial resolution, we can still clearly see the defocus effect and easily pinpoint the best-focused plane.

We also examine the simulated axial intensity profiles of the point sources placed between 300 mm and 480 mm with a separation of 30 mm, as shown in Fig. S10. This depth configuration matches the seven equally-spaced depth planes used in our training dataset capture. We see that the amount of separation is well below the FWHM, however, we can expect that the defocus blur is still observed within the depth range of 180 mm.

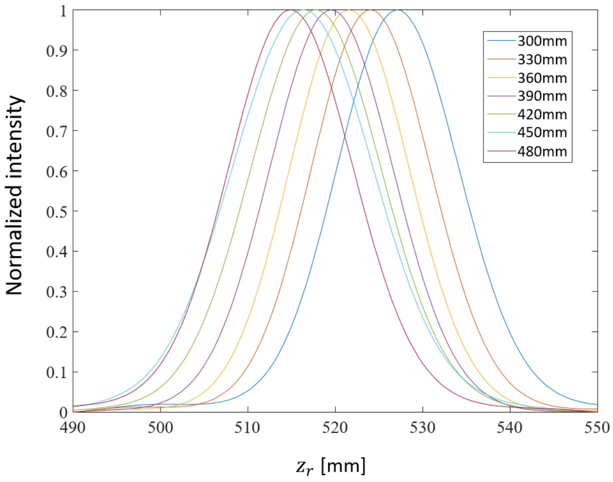

Fig. S10 Simulated axial intensity profiles of the point sources placed with a physical separation of 30 mm.

We found that the axial resolution is not an ideal measure of the 3D camera; therefore, proper quantification of depth resolution should be investigated. This will require the consideration of the amount of required blur for daily-use 3D cameras and an understanding of the depth perception of the camera or display users. We believe studying such issues is beyond the scope of our paper, and we leave further investigation as future work.

2.3 Field of view

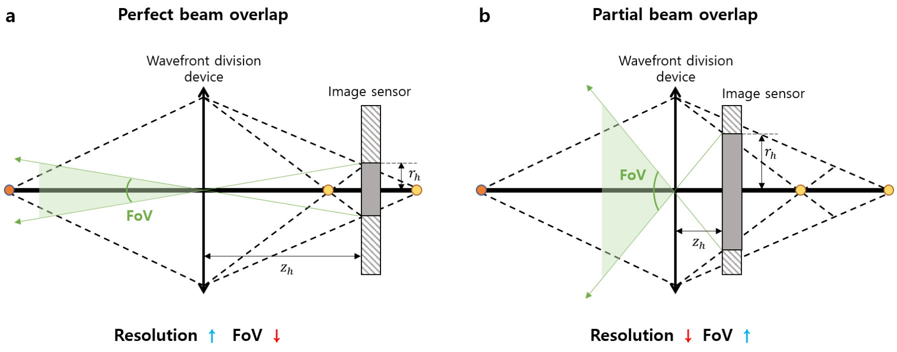

Fig. S11 Comparisons of beam overlap conditions. a Perfect beam overlap condition provides high lateral resolution but reduced FoV. **b** Partial beam overlap expands FoV at the expense of lateral resolution. Grayed areas indicate the beam overlap regions.

We optimize the configuration of the incoherent holographic camera for capturing life-sized objects by enlarging the FoV. The primary way to achieve the large FoV in GP-SIDH systems is to exploit the partial beam overlap condition as shown in Fig. S11b in contrast to the perfect beam overlap condition that is typically employed in FINCH systems as shown in Fig. S11a. The FoV of both configurations is given as

$$\text{FoV} = 2\arctan(\frac{r_h}{z_h}) \tag{S33}$$

where r_h is the hologram radius and z_h is the distance between wavefront division device and image sensor. We can see that the partial beam overlap condition in the GP-SIDH systems provides the reduced z_h and increased r_h, resulting in the expanded FoV. The partial beam overlap is not an ideal condition because it leads to the degradation of the lateral resolution [9] However, considering that our main goal is to capture life-size objects and that there is an inevitable trade-off between FoV and lateral resolution, we decide to increase the FoV at the expense of the lateral resolution.

Although it looks as if the expansion of FoV can be simply achieved by placing the image sensor closer to the wavefront division device, the actual modification from the system in Fig. S11a to the system in Fig. S11b can be made only if two important conditions are satisfied:

Condition 1 Reducing z_h and increasing r_h are physically plausible.
Condition 2 The captured holograms should provide enough lateral and axial resolution. Otherwise, there is no benefit of using incoherent holographic cameras over conventional 2D cameras.

We found that the GP lens plays an important role in fulfilling those conditions. Regarding *Condition 1*, the GP lens can easily satisfies this condition: (1) the GP lens works with the transmission geometry unlike the LCoS SLM, which typically works with the reflection geometry, therefore we can reduce z_h down to a few millimeters, and (2) GP can be fabricated large enough so that the aperture size of the GP lens does not limit the hologram size r_h.

The validation of *Condition 2* requires more careful consideration of the focus values of the wavefront division devices. In the following, we show that the positive and negative focal lengths of the GP lens is a key property to achieve a reasonable lateral and axial resolution for the system configuration in Fig. S11b. As shown in Sec. 2.1, the hologram radius is typically limited by the sensor size; therefore, the reconstruction distance z_r is the crucial factor that determines the lateral and axial resolution of the system. The formulation of z_r in Eq. (S22) is generalized for two arbitrary focal lengths f_1 and f_2 as follows [11]:

$$z_r = \frac{(f_1 z_s - z_h z_s + f_1 z_h)(f_2 z_s - z_h z_s + f_2 z_h)}{z_s^2 (f_1 - f_2)}. \tag{S34}$$

We examine the best resolution condition based on three representative types of focal length pairs induced by existing wavefront division devices as shown in Figs. S12a-c. The first type (Fig. S12a) represents birefringence lenses [12] and the optical power difference between f_1 and f_2 are typically within 10%. The second type (Fig. S12b) corresponds to LCoS SLMs [13] and they have low f-numbers due to the small diffraction angle and aperture size. The third type (Fig. S12c) represents the GP lens used in our GP-SIDH system. They produce the negative and positive focal lengths with the same magnitude.

Focus Type	Wavefront division device	Setting values (mm)	z_r (mm)	Lateral resolution (mm)	Axial resolution (mm)
f_1, f_2	Birefringence lens	1100, 900	5074	22.8	4304.2
∞, f	LCoS SLM	∞, 1000	1033	4.6	839.5
$-f, f$	**GP lens**	-1000, 1000	520	2.5	444.1

Table S1 Tentative lateral and axial resolutions provided by various wavefront division devices.

As the parametric space can be huge in Eq. (S34), we fix z_h to 8 mm, which is a reasonable setting to achieve the large FoV in the configuration presented Fig. S11b. We also set the object position z_s to 390 mm, which is the

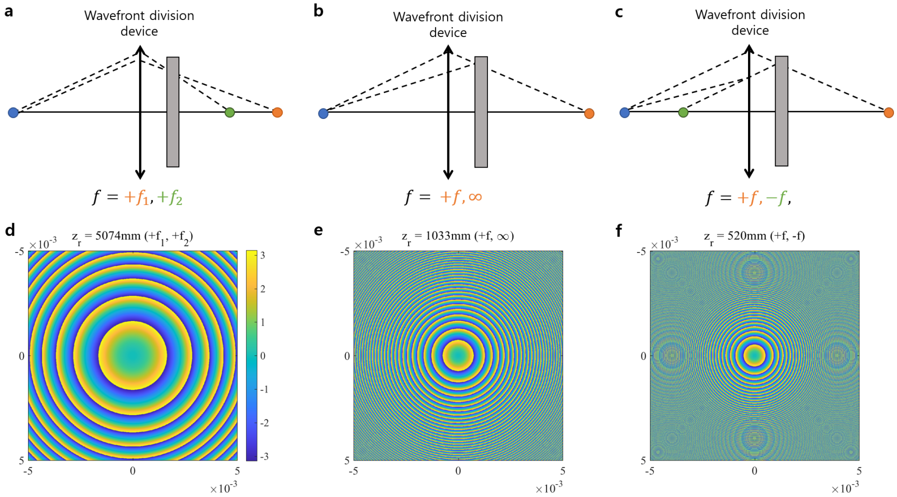

Fig. S12 Types of focal length pairs and corresponding simulated holograms a Focal lengths have the same signs. **b** Transmissive beam and converging beam modulated with a positive focal length are generated. **c** Positive and negative focal lengths with the same magnitude are generated. **d, e, f** Simulated holograms for focal length pairs in **a, b, c**, respectively.

center position of our target depth range [300 mm, 480 mm] of DeepIHC. The focal lengths of the birefringence lens and LCoS SLM are set to have similar focal power with respect the GP lens. Under these conditions, we calculate the lateral and axial resolutions, as shown in Table S1. Although the exact values of the resolutions can vary depending on the focal length value setting, Table S1 indicates that GP lens is a good direction to achieve the increased lateral and axial resolution when the system is optimized to have the large FoV. We also visually examine the example holograms for three cases in Figs. S12d-f. The holograms show that higher spatial frequencies can be captured using the GP lens compared to the cases when birefringence lens or LCoS SLMs is used, indicating that higher lateral and axial resolutions can be obtained with the GP lens.

3 Supporting results

3.1 Hologram data of the human face

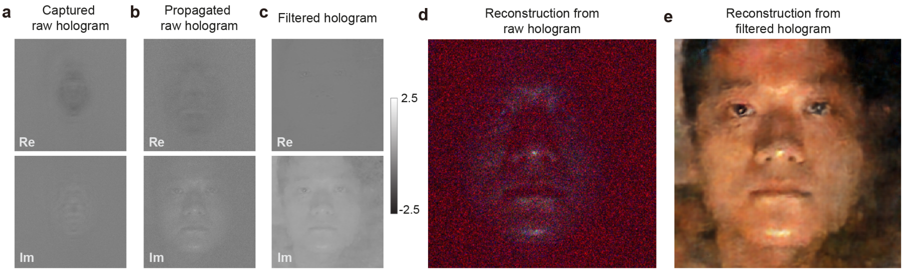

Fig. S13 Hologram data of the human face in Fig. 1. a Raw hologram acquired by GP-SIDH. **b** Raw hologram propagated to the central plane of the object. **c** Filtered hologram inferred by the neural network. The real and imaginary parts are only shown for the green channel in all hologram data. **d** Image of the human face reconstructed from the raw hologram in **b**. **e** Image of the human face reconstructed from the filtered hologram in **c**.

3.2 Validation of denoising images with unseen objects

We test whether objects that do not appear in the training dataset can be denoised properly during the validation stage as shown in Fig. S14. We examine that all our training images selected from the DIV2K dataset do not contain object images similar to the letters 'POLICE', the ancient statue, the red car, and the yellow helmet that appear in the target validation images in Fig. S14a. However, we can see that DeepIHC still provides the average enhancement of PSNR of 10.6 dB in the reconstructed images of the filtered hologram in Fig. S14c over the reconstructed images of the raw hologram in Fig. S14b.

3.3 Effect of conditioning the phase map

Given that the image loss is computed only at the target focus plane, it would be interesting to test whether providing depth information is helpful to the network. An additional depth constraint is tested in the form of complex field loss [14]. For the captured hologram for the target image I_i at d_i, it is assumed that the phase of the hologram at depth d_i is uniform and has an offset proportional to the distance from the central plane. The ground-truth amplitude A_i and phase ϕ_i are set as follows:

$$A_i = \sqrt{I_i},\ \phi_{i,\lambda} = \frac{\pi(d_i - d_c)}{\lambda}. \tag{S35}$$

For $H_{recon} = \tilde{A}e^{i\tilde{\phi}}$, we additionally consider the complex field loss by following the approach in Ref. [14].

$$l_{comp} = \|\tilde{A} - Ae^{i[\delta(\tilde{\phi},\phi) - \bar{\delta}(\tilde{\phi},\phi)]}\|_2 \tag{S36}$$

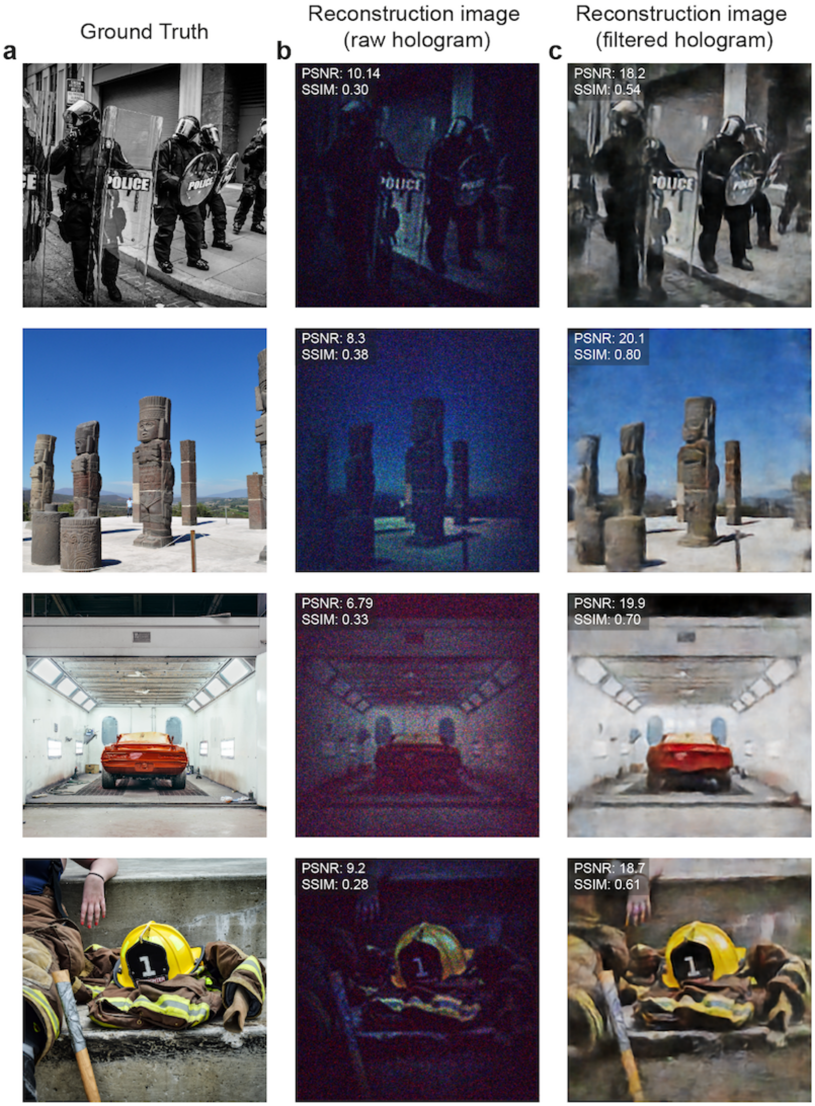

Fig. S14 Validation of denoising images with unseen objects. a Validation target images that contain objects which did not appear in the training dataset. **b** Images reconstructed at the corresponding target object depths from the raw holograms. **c** Images reconstructed from the filtered holograms.

where $\delta(\tilde{\phi}, \phi) = \text{atan2}[\sin(\tilde{\phi} - \phi), \cos(\tilde{\phi} - \phi)]$ and $\bar{\cdot}$ denotes the mean.

Figs. S15d-f present the reconstructed images obtained by using the network trained with the additional complex field loss. A slight degradation in the image quality is observed for the flat 2D object in Fig. S15d, which is acceptable. However, the network fails to filter the hologram for the miniature house scene and generates strange artifacts in Figs. S15e and S15f. Therefore, it can

be inferred that leaving the phase unconstrained leads to better handling of the multidepth configuration. However, the possibility that the complex field loss might not be adequate to handle incoherent holograms must not be excluded. Therefore, better training strategies should be investigated in future works.

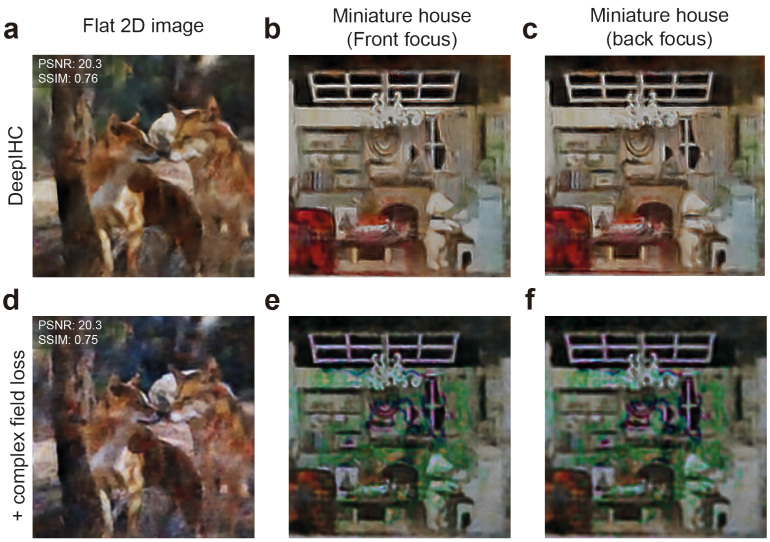

Fig. S15 Effect of conditioning the phase map during the training process. Reconstructed focal images derived from the holograms filtered by our proposed neural network for **a** a flat image object and **b, c** the miniature house scene. Reconstructed focal images derived from the holograms processed by a neural network trained with the complex field loss for **d** a flat image object and **e, f** the miniature house scene. Only the neural network of DeepIHC provides an accurate reproduction of the 3D object.

3.4 Reference photograph of the music box

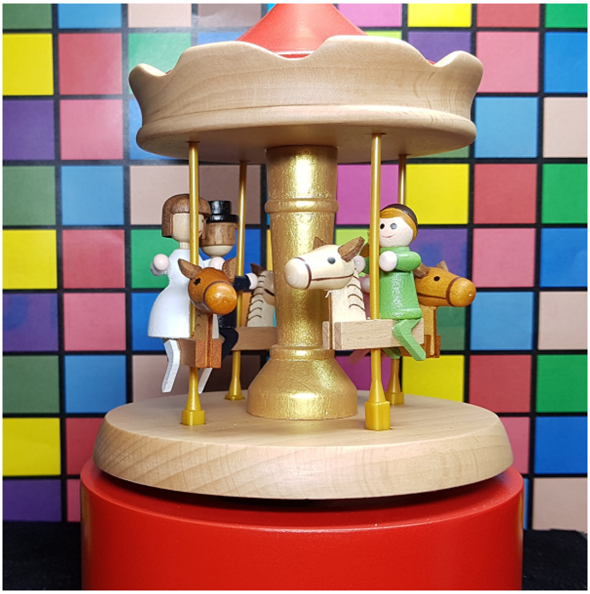

Fig. S16 Reference photograph of the music box.

4 Pseudocodes

Algorithm 1 System Calibration

D: number of depth planes = 7
d_i: depth position of the i-th depth plane
d-ASM(H, d): depth-corrected angular spectrum method that propagates hologram H by distance d
$[p_d^1, p_d^2, p_d^3, p_d^4]$: positions of four corner points of the frame to display reference images at depth d
$[q_d^1, q_d^2, q_d^3, q_d^4]$: positions of four points in the reconstructed image at depth d as indicated in Fig. S3b
$[c_d^1, c_d^2, c_d^3, c_d^4]$: positions of four outermost corners of the grid pattern in the reconstructed image at depth d

for $d \leftarrow d_1$ to d_D **do**
  Step 1: Place the tablet monitor at depth d
  Step 2: Display the grid pattern image inside the frame $[p_d^1, p_d^2, p_d^3, p_d^4]$
  Step 3: Capture a hologram H_d
  Step 4: $H_{recon} \leftarrow$ d-ASM(H_d, d)
  Step 5: $I_{recon} \leftarrow |H_{recon}|^2$
  Step 6: Detect the positions $[q_d^1, q_d^2, q_d^3, q_d^4]$ of four points in I_{recon} as indicated in Fig. S3b
  Step 7: Extrapolate $[q_d^1, q_d^2, q_d^3, q_d^4]$ to obtain the positions $[c_d^1, c_d^2, c_d^3, c_d^4]$ of the four outermost corners of the grid pattern
  Step 8: If the area spanned by $[c_d^1, c_d^2, c_d^3, c_d^4]$ is notably deviated from 600×600 pixels, adjust $[p_d^1, p_d^2, p_d^3, p_d^4]$ and repeat Steps 2 to 7
  Step 9: Store warping transformation W_d from $[c_d^1, c_d^2, c_d^3, c_d^4]$ to [(0,0), (0, 600), (600, 0), (600, 600)]
end for

Algorithm 2 Dataset Capture

R: total number of reference images = 500
R_1: number of reference images belong to Set 1 = 250
w, h: width and height of reference images
w_b, h_b: width and height of null region
D: number of depth planes = 7
d_i: depth position of the i-th depth plane
$[p_d^1, p_d^2, p_d^3, p_d^4]$: four corner points of the frame to display reference images at depth d. They are determined in Algorithm 1
$I_{c,i}$ for $i = 0, 1, 2, 3$: phase-shifting images as defined in Eqs. S5-8

# Null Region Insertion For Set 3
for $r \leftarrow R_1 + 1$ to R **do**
  Step 1: Load a reference image I_r
  Step 2: Randomly pick $w_b \in [0.25w, 0.5w]$
  Step 3: Randomly pick $h_b \in [0.25h, 0.5h]$
  Step 4: Insert rectangular null region with a size of w_b and h_b inside I_r
  Step 5: Update I_r
end for

# Dataset Acquisition
for $d \leftarrow d_1$ to d_D **do**
  Step 1: Place the tablet monitor at d
  for $r \leftarrow 1$ to R **do**
    Step 2: Display a reference image I_r inside the frame $[p_d^1, p_d^2, p_d^3, p_d^4]$
    Step 3: Capture a raw camera image I_{raw}
    Step 4: $I_{raw,0} \leftarrow I_{raw}(x = odd, y = odd)$
    Step 5: $I_{raw,1} \leftarrow I_{raw}(x = odd, y = even)$
    Step 6: $I_{raw,2} \leftarrow I_{raw}(x = even, y = even)$
    Step 7: $I_{raw,3} \leftarrow I_{raw}(x = even, y = odd)$
    Step 8: $I_{c,i} \leftarrow$ demosaic($I_{raw,i}$, 'RGGB') for $i = 0, 1, 2, 3$
    Step 9: $H \leftarrow (I_{c,0} - I_{c,2}) - i(I_{c,1} - I_{c,3})$
    if $r <= R_1$ **then**
      Step 10: Save H to Set 1
    else
      Step 11: Save H to Set 3
    end if
  end for
end for

Algorithm 3 Neural Network Training

E: number of training epochs = 400
N: number of training images = 5250
d_c: depth of the central plane = 39 cm
ROI: region of interest = 720 × 720
S: subpatch region among 5×5 grid in the hologram domain
S': subpatch region in the reconstruction domain corresponding to S
trainType(n): function assigning dataset type of the current training data. Set 2 is augmented from captured holograms in Set 1
sample(T, n): function sampling hologram from Set 1 for T = 1, 2 and from Set 3 for T = 3. Returns hologram and corresponding depth
d-ASM(H, d): depth-corrected angular spectrum method that propagates hologram H by distance d
augment(H, S, d): function preserving the subpatch S of H and replacing the remaining region with a hologram randomly chosen from Set 1 and captured at other than d
$M(\cdot;\ \theta)$: hologram filtering neural network model with parameters θ
ASM(H, d): conventional angular spectrum method that propagates hologram H by distance d
$W_d(\cdot)$: warping transformation at depth d acquired in Algorithm 1
$l_{pcp}(\cdot)$: perceptual loss function

for $e \leftarrow 1$ to E **do**
  for $n \leftarrow 1$ to N **do**
    Step 1: $T \leftarrow$ trainType(n)
    Step 2: $H_n, d_n \leftarrow$ sample(T, n) ▷ Random shuffle used
    Step 3: Retrieve the corresponding reference image I_n for H_n
    Step 4: $H_{center} \leftarrow$ d-ASM(H_n, d_c)
    if $T ==$ 'Set 2' **then**
      Step 5: Randomly pick subpatch S.
      Step 6: $H_{center} \leftarrow$ augment(H_{center}, S, d_n)
    end if
    Step 7: $H_{center} \leftarrow$ crop(H_{center}, ROI)
    Step 8: $H_{out} \leftarrow M(H_{center};\ \theta)$
    Step 9: $H_{recon} \leftarrow$ ASM($H_{out}, d_n - d_c$)
    Step 10: $H_{recon} \leftarrow W_{d_n}(H_{recon})$
    Step 11: $I_{recon} \leftarrow |H_{recon}|^2$
    if $T ==$ 'Set 2' **then**
      Step 12: $I_{recon} \leftarrow$ crop(I_{recon}, S')
      Step 13: $I_n \leftarrow$ crop(I_n, S')
    end if
    Step 14: loss = $l_{pcp}(I_{recon}, I_n)$
    Step 15: Perform backpropagation and update model parameter θ
  end for
end for

Algorithm 4 Neural Network Inference

d_c: depth of the central plane = 39 cm
ROI: region of interest = 720 × 720
$M(\cdot;\ \theta)$: hologram filtering neural network model with parameters θ
d-ASM(H, d): depth-corrected angular spectrum method that propagates hologram H by distance d

Step 1: Capture a hologram H
Step 2: $H_{center} \leftarrow$ d-ASM(H, d_c)
Step 3: $H_{center} \leftarrow$ crop(H_{center}, ROI)
Step 4: $H_{filtered} \leftarrow M(H_{center};\ \theta)$

Algorithm 5 Examination of Filtered Hologram

$H_{filtered}$: filtered hologram acquired by DeepIHC
d_{target}: target examination depth

Step 1: $H_{recon} \leftarrow \text{ASM}(H_{filtered}, d_{target} - d_c)$
Step 2: $I_{recon} \leftarrow |H_{recon}|^2$
Step 3: Examine focal stack image I_{recon}

Algorithm 6 Display of Filtered Hologram

$H_{filtered}$: filtered hologram acquired by DeepIHC
rescale$(\cdot, \textit{range})$: rescale function mapping input values to *range*

Step 1: $P_{proj} \leftarrow \mathbf{Re}(H_{filtered}) + |H_{filtered}|$
Step 2: $P_{proj} \leftarrow \text{rescale}(P_{proj}, [0, 255])$
Step 3: Display P_{proj} on the panel.

References

[1] Roux, F. S. Geometric phase lens. *JOSA A* **23** (2), 476–482 (2006). https://doi.org/10.1364/JOSAA.23.000476 .

[2] Choi, K. *et al.* Compact self-interference incoherent digital holographic camera system with real-time operation. *Opt. Express* **27** (4), 4818–4833 (2019). https://doi.org/10.1364/OE.27.004818 .

[3] Yousefzadeh, C., Jamali, A., McGinty, C. & Bos, P. J. Achromatic limits of pancharatnam phase lenses. *Appl. Opt.* **57** (5), 1151–1158 (2018). https://doi.org/10.1364/AO.57.001151 .

[4] Kim, J. *et al.* Fabrication of ideal geometric-phase holograms with arbitrary wavefronts. *Optica* **2** (11), 958–964 (2015). https://doi.org/10.1364/OPTICA.2.000958 .

[5] Peng, Y., Choi, S., Padmanaban, N. & Wetzstein, G. Neural holography with camera-in-the-loop training. *ACM Trans. Graph.* **39** (6) (2020). https://doi.org/10.1145/3414685.3417802 .

[6] Choi, S., Kim, J., Peng, Y. & Wetzstein, G. Optimizing image quality for holographic near-eye displays with michelson holography. *Optica* **8** (2), 143–146 (2021). https://doi.org/10.1364/OPTICA.410622 .

[7] Choi, S., Gopakumar, M., Peng, Y., Kim, J. & Wetzstein, G. Neural 3d holography: Learning accurate wave propagation models for 3d holographic virtual and augmented reality displays. *ACM Trans. Graph. (SIGGRAPH Asia)* (2021). https://doi.org/10.1145/3478513.3480542 .

[8] Chakravarthula, P., Tseng, E., Srivastava, T., Fuchs, H. & Heide, F. Learned hardware-in-the-loop phase retrieval for holographic near-eye displays. *ACM Transactions on Graphics (TOG)* **39** (6), 186 (2020). https://doi.org/10.1145/3414685.3417846 .

[9] Rosen, J., Siegel, N. & Brooker, G. Theoretical and experimental demonstration of resolution beyond the rayleigh limit by finch fluorescence microscopic imaging. *Opt. Express* **19** (27), 26249–26268 (2011). https://doi.org/10.1364/OE.19.026249 .

[10] Siegel, N. & Brooker, G. Improved axial resolution of finch fluorescence microscopy when combined with spinning disk confocal microscopy. *Optics express* **22** (19), 22298–22307 (2014). https://doi.org/10.1364/OE.22.022298 .

[11] Katz, B. & Rosen, J. Super-resolution in incoherent optical imaging using synthetic aperture with fresnel elements. *Opt. Express* **18** (2), 962–972

(2010). https://doi.org/10.1364/OE.18.000962 .

[12] Siegel, N., Lupashin, V., Storrie, B. & Brooker, G. High-magnification super-resolution finch microscopy using birefringent crystal lens interferometers. *Nature photonics* **10** (12), 802–808 (2016). https://doi.org/10.1038/nphoton.2016.207 .

[13] Rosen, J. & Brooker, G. Digital spatially incoherent fresnel holography. *Opt. Lett.* **32** (8), 912–914 (2007). https://doi.org/10.1364/OL.32.000912 .

[14] Shi, L., Li, B., Kim, C., Kellnhofer, P. & Matusik, W. Towards real-time photorealistic 3d holography with deep neural networks. *Nature* **592** (2021). https://doi.org/10.1038/s41586-020-03152-0 .
