# Supplementary material for: Deep learning-based incoherent holographic camera enabling acquisition of real-world holograms for holographic streaming system
Source: Nat Commun. 2023 Jun 14;14:3534. doi: 10.1038/s41467-023-39329-0 (PMC10267150; doi:10.1038/s41467-023-39329-0)
Supplement: Supplementary file 3 — Description to Additional Supplementary Information [file 41467_2023_39329_MOESM3_ESM.pdf]

Description of Additional Supplementary Files

Supplementary video 1: Holographic streaming system demonstration for a static object

Supplementary video 2: Holographic streaming system demonstration for a moving object

Supplementary video 3: Video footage of real-time human face capture demonstration

Supplementary video 4: Reconstructed videos of human face holograms at various depths
